# The role of endoscopic management and adjuvant topical therapy for upper tract urothelial cancer

**DOI:** 10.3389/fruro.2022.916259

**Published:** 2022-09-19

**Authors:** Austin L. Chien, Kevin J. Chua, Sai Krishnaraya Doppalapudi, Saum Ghodoussipour

**Affiliations:** 1 Section of Urologic Oncology, Rutgers Cancer Institute of New Jersey, New Brunswick, NJ, United States,; 2 Division of Urology, Department of Surgery, Rutgers Robert Wood Johnson Medical School, New Brunswick, NJ, United States

**Keywords:** upper tract urothelial carcinoma, upper tract urothelial cancer, ureteral cancer, ureteral cancer endoscopic treatment, ureteroscope, instillation, kidney sparing procedures (KSP), nephron sparing

## Abstract

Upper tract urothelial carcinoma (UTUC) has traditionally been managed with radical nephroureterectomy, and while this approach remains the gold standard for high-risk disease, endoscopic, kidney-sparing management has increasingly been adopted for low-risk disease as it preserves kidney function without compromising oncologic outcomes. Ureteroscopy and percutaneous renal access not only provide diagnoses by tumor visualization and biopsy, but also enable treatment with electrocautery or laser ablation. Several modalities exist for laser ablative treatments including thulium:YAG, neodymium:YAG, holmium:YAG, and combinations of the preceding. Furthermore, due to high recurrence rates after endoscopic management, adjuvant intracavitary instillation of various agents such as mitomycin C and bacillus Calmette-Guerin have been used given benefits seen in non-muscle invasive urothelial bladder cancer. Other formulations also being studied include gemcitabine, anthracyclines, and immunotherapies. More recently, Jelmyto, a mitomycin reverse thermal gel, has been developed to allow for adequate drug delivery time and potency since urine flow could otherwise dilute and washout topical therapy. In this article, the authors review techniques, indications, best practices, and areas of current investigation in endoscopic management and adjuvant topical therapy for UTUC.

## Introduction

Upper tract urothelial carcinomas (UTUCs) encompass any malignancies arising from the urothelium of the urinary tract spanning the renal calyces most proximally, to the ureterovesical junction most distally. This designation includes, most commonly, urothelial cancer, as well as rarer nonurothelial cancers such as adenocarcinoma and squamous cell carcinoma (1). Presentation is unilateral in 95% of patients, with multifocal manifestations seen in 10% to 20% of cases. UTUC has an annual incidence of 1–2 cases per 100,000 inhabitants in the United States. It accounts for 5–7% of all renal tumors and 5–10% of all urothelial tumors, with a rate of incidence that has steadily risen over the past several decades with improved diagnostic approaches (1, 2). Incidence of more advanced stages of disease have also been seen to increase since the early 21^st^ century (3). Greatest incidence is in patients age 70 to 90, with a mean age at diagnosis of 73. Associations of incidence rates to gender and race are equivocal. While earlier reporting has suggested that incidence is greater among males and African-Americans (1, 2), recent reviews of the literature have found mixed reports across both variables (4, 5). Balkan nations are known to have an especially increased prevalence compared to other populations, with UTUC comprising 40% of renal tumors in Balkan countries (2). Survival rates for overall disease, carcinoma *in situ*, localized disease, disease with regional spread, and disease with distant metastasis is 75%, 95%, 88.9%, 62.5%, and 16.5%, respectively (1). Cancer-specific mortality has been shown to be increased in black males compared to white males (7.4% vs 4.9%), in women compared to men (6.1% vs 4.4%) (1), and in patients with rural area residence status compared to urban area status (26.7% vs 15.7%) (6).

**FIGURE 1:**
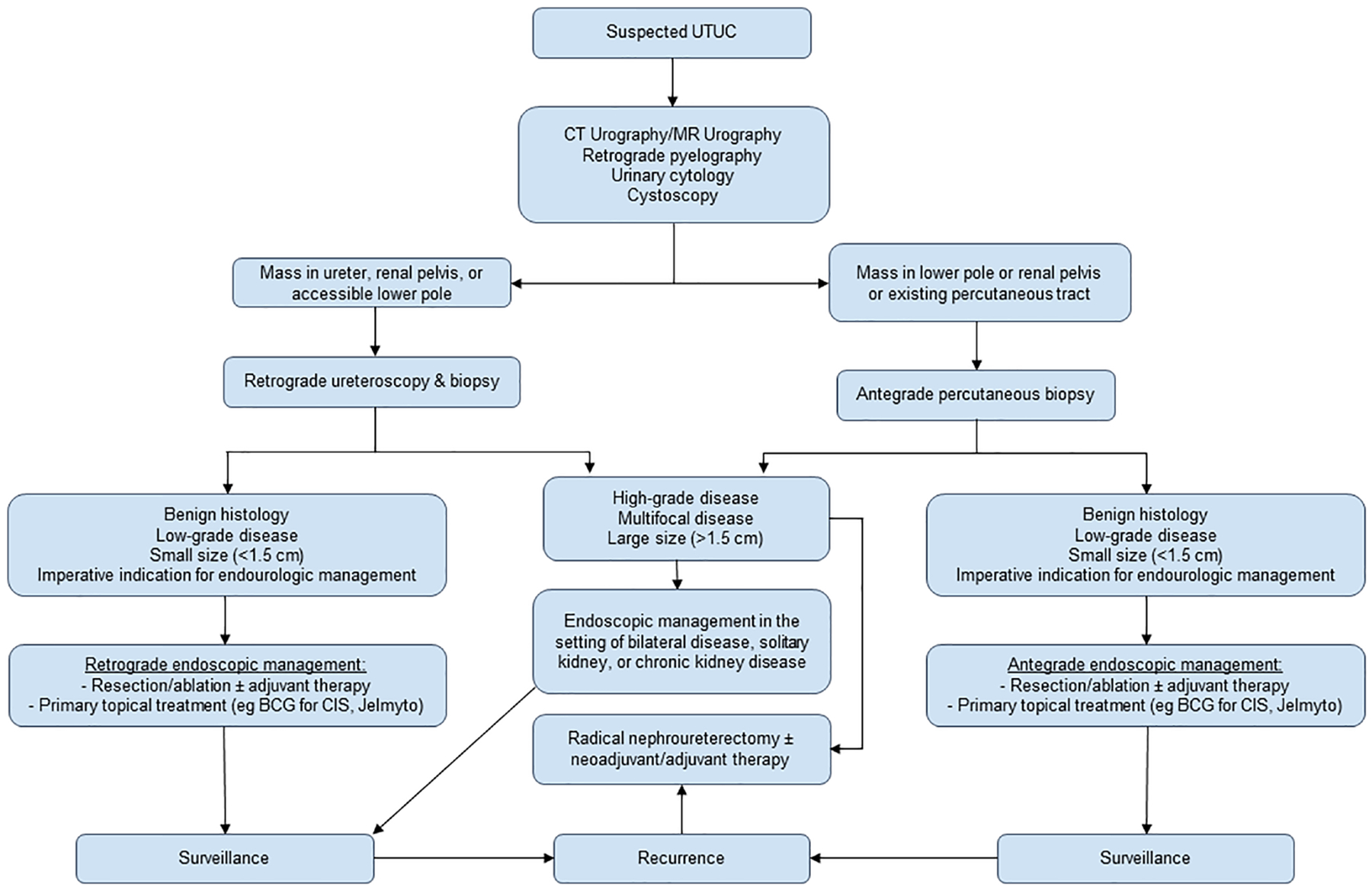
Recommended UTUC treatment algorithm.

The gold standard of treatment for large, high-grade, or invasive UTUC is radical nephroureterectomy with excision of the ipsilateral bladder cuff (7, 8). However, radical nephroureterectomy is subject to complications such as loss of renal function, prolonged length of hospital stay, and infection at rates of 30–40% (9, 10). Thus, endourologic kidney- and nephron-sparing management are now employed with increasing indications. Percutaneous and ureteroscopic management of UTUC have been established as safe, efficient treatment methods for UTUC with appropriate patient selection. Renal preservation rates with ureteroscopic management are approximately 71%, with cancer-specific survival above 90% in contemporary studies (11). Complications are relatively minor, most commonly consisting of ureteral stricture; or minor pain, bleeding, or fever (12). However, endourologic treatment strategies suffer from relatively high rates of disease recurrence in the upper or lower tract, which can range from 15% to 90% depending on patient follow-up and disease characteristics (13). Elective indications for endourologic management of UTUC include low-grade disease, papillary architecture, tumor size <1.5 cm, unifocal tumor presence, and absence of invasive disease on imaging. Imperative indications include poor surgical candidacy, a solitary kidney (whether by anatomy or limited renal function), chronic renal disease and impairment, and bilateral tumors (8). In addition to resection or ablative endoscopic techniques, intracavitary chemotherapy instillations are an increasing area of investigation and treatment development. Figure 1 provides a summative algorithm outlining recommended management.

In this focused review, we review techniques, indications, best practices, and areas of current investigation in endourologic management and adjuvant topical therapy for UTUC. A literature search in PubMed was conducted for the following search terms in conjunction with upper tract urothelial carcinoma or UTUC: endoscopy, ureteroscopy, percutaneous, retrograde, instillation, topical, diagnostic ureteroscopy, percutaneous resection/ablation, and retrograde resection/ablation. All returned articles were investigated. Inclusion criteria for reference literature were relation of the text to endoscopic or non-invasive UTUC management and publication date from 2000 to 2022. Additional articles were subsequently retrieved from the bibliographies of already-examined literature, and select references published prior to 2000 have been included as needed for contextual explanation of techniques or history. Articles focusing primarily on nephroureterectomy, open surgery, or laparoscopic surgical techniques and their indications were excluded.

## Diagnostic ureteroscopy

Endourologic interventions in UTUC begin at the initial staging processes. While radiologic imaging is an essential component of UTUC diagnosis and workup, radiological suspicion of UTUC alone is poorly correlated with the findings at ureteroscopy. Though multidetector computed tomography (CT) urography is the standard for UTUC imaging modalities, routine flexible ureteroscopy has been shown to reduce misdiagnosis of UTUC by CT urography from 15.5% to 2.1% (14). UTUC-positive ureteroscopy findings have been seen with 55.6% and 48% of patients with unlikely and negative CT urography, respectively. Positive CT urography findings have additionally differed from final positive ureteroscopy findings in dimensions, number, or site in 42.1% of cases (15). Appearance on retrograde pyelography and surgical pathologic grade have shown an overall concordance of 75%, with greater correlation between imaging interpretation and surgical pathology grading in lower grade disease, suggestive of an overall pattern of pyelography underestimating tumor grade (16). These results further highlight the utility of ureteroscopic visualization and biopsy for UTUC diagnosis.

Though ureteroscopic diagnostics may obviate the need for surgical biopsy, current evidence suggests that histopathologic assessment is still necessary for accurate tumor characterization. Diagnostic ureteroscopy often suffers from inadequate tissue yield, frequently leading to understaging of the pathology on ureteroscopy compared to final surgical pathology (16, 17). Visualization of UTUC on ureteroscopy can have predictive value, with sessile tumor appearance associated with high-stage disease (18). Diagnostic nomograms that incorporate imaging, ureteroscopic appearance, and pathologic grading to predict surgical treatment risks and outcomes are an area of active investigation (19–21).

While ureteroscopy has clear value for visualization and biopsy of UTUC, the potential for intraoperative seeding and increased rates of post-resection recurrence has been much debated. In 2010, Ishikawa et al. evaluated a sample of 208 patients, of whom 55 underwent diagnostic ureteroscopy prior to nephroureterectomy, and found no significant difference in intravesical rates of recurrence (log-rank test p-value=0.972). 2-year recurrence-free survival rates were similar between the two study arms (60.0% in the ureteroscopy group vs 58.7% in the controls), as were cancer-specific survival rates (88.3% vs 78.1%, respectively, with log rank test p-value=0.0687) (22). Conversely, a 2016 study by Sankin et al. of 144 patients undergoing ureteroscopy prior to nephroureterecomy and 57 patients bypassing ureteroscopy to undergo nephroureterectomy found significant association between diagnostic ureteroscopy prior to nephroureterectomy and intravesical recurrence (hazard ratio 2.58; 95% CI 1.47, 4.54; p-value=0.001). This increased risk of recurrence may have minimal impact on the patient course, however, as Sankin et al. found no associations between diagnostic ureteroscopy and cancer-specific survival, metastasis-free survival, or overall survival (23). A 2017 systematic review and meta-analysis by Marchioni et al. found a statistically significant association between ureteroscopy prior to radical nephroureterectomy and intravesical recurrence across a pooled sample of 6 studies (HR 1.56, 95% CI 1.33–1.88; P < 0.001) (24). Recent work by Douglawi et al. supported the association between ureteroscopy and recurrence. In a sample of 143 patients who had radical nephroureterectomy (104 of whom underwent prior ureteroscopy), 30.8% of patients who underwent ureteroscopy experienced recurrence compared to 7.7% of patients without ureteroscopy (p=0.02). Time to recurrence was also correlated, with ureteroscopy patients and non-ureteroscopy patients having a median time to recurrence of 9.0 and 12.1 months, respectively. Follow-up multivariable analysis confirmed an increased rate of bladder recurrence in patients with ureteroscopy prior to nephroureterectomy (HR 5.6, P <.004). Of note, however, was the finding that patients whose ureteroscopy employed a ureteral access sheath (26 of 104) had a recurrence rate of 11.5%, while those without an access sheath had a rate of 39.7% (p=0.01). The use of a ureteral access sheath appeared to mitigate the increased rate of bladder recurrence in ureteroscopy patients, with multivariable analysis showing no significant differences between access sheath patients and those who never underwent ureteroscopy (HR 1.3, p 0.76) (25). Further investigation will be needed to better characterize this potential association, as well as determine its potential origins, other associated variables, and implications for UTUC management.

In light of the utility of ureteroscopic techniques for diagnosis and characterization of UTUC, several imaging and biopsy devices or techniques have been developed (Table 1). Endoluminal ultrasonography (ELUS) has shown utility in select cases for UTUC staging. Farnum et al. employed a mechanical radial scanning ultrasound at 20 MHz in B-mode with a 5F probe to compare ultrasound staging with findings on surgical pathology taken after nephroureterectomy. ELUS was found to have a positive predictive value (PPV) of 76.2% in patients with non-muscle invasive UTUC, and 16.7% in patients with invasive disease. These PPV results, particularly for pT2-pT3 disease, highlight the primary limitation of ELUS, and further work remains to be done before it is adopted for widespread use (26). Narrow-band imaging (NBI) is an endoscopic visualization technique that consists of filtering white light to into blue and green wavelengths that better penetrate mucosa and are absorbed by hemoglobin, thereby forming a more detailed view of mucosal tissue and blood vessels. NBI has been in use for evaluation of the gastrointestinal tract (34) and has demonstrated improved accuracy for the diagnosis of bladder tumors leading to adoption under urologic guidelines (35). Literature specifically exploring NBI with regards to UTUC is limited, with one study to date that assessed 27 patients. The authors reported subjective improvement in visualization, and detected an additional five tumors in four patients as well as increased tumor width on NBI when compared to white light visualization (27). Though data is limited, broader adoption of NBI for UTUC evaluation is feasible given the promising findings and its pre-existing use by practicing urologists. Photodynamic diagnosis (PDD), also known as fluorescent cystoscopy or blue-light cystoscopy, is an imaging modality that employs a photosensitizing agent which is injected into the intravesical cavity (28) or administered orally (36). During endoscopy, blue light (375–445 nm) is shone causing the agent to fluoresce. PDD has shown promising results. A systematic review and meta-analysis by Qiangzhao et al. across six studies with 289 tumors found that PDD could distinguish UTUC from noncancerous sites at a sensitivity of 0.96 and a specificity of 0.86. The authors also found that PDD improved the additional detection rate of UTUC compared with white-light ureteroscopy (RR 0.16, 95% CI 0.07–0.34 p-value=0.000) (28). Though PDD has demonstrated favorable outcomes, further validation remains to be seen, and clinically available systems may not demonstrate equivalent findings (37).

Other techniques have been developed to enable real-time intraoperative histological characterization of tumor grade and stage. Optical coherence tomography (OCT) uses back-scattered light to produce micrometer-scale resolution cross-sectional images, analogous to ultrasound’s use of back-reflected sound waves to produce imaging. OCT has been investigated in porcine and human studies and has shown improved capacity to distinguish ureteral wall layers when compared to ELUS, as well as capability to differentiate between noninvasive versus invasive tumors and low- versus high-grade tumors. Currently available systems are limited, however, to a maximal diameter of 10 mm and a depth of 2 mm due to light scattering (29, 38). Diagnostic confocal laser endomicroscopy (CLE) similarly provides real-time high-resolution imaging using a 488nm low-energy laser which scans tissue stained with fluorescein. An intraoperative photosensitizer excites the stained tissue, which emits light that is filtered such that only in-focus light is recorded, leading to high resolution intraoperative imaging and potential tumor grading on par with histology (30, 31). While optical diagnostic techniques such as OCT and CLE show promising initial results, more research will be needed prior to greater incorporation in the clinical setting.

Approaches to biopsy can include use of a variety of tools, such as 3-F cup biopsy forceps, grasping forceps, tipless nitinol baskets, and brushes. While 3-F forceps are a commonly used biopsy tool, recent studies have demonstrated greater quality specimen on capture *via* use of backloaded cup forceps and nitinol baskets (39–41). Image-enhancement techniques have also been developed for ureteroscopic devices, with the development of digital ureteroscopy enabling improved endoscopic viewing of the upper tract (32). Digital ureteroscopy has demonstrated improved image quality compared to fiber-optic ureteroscopy, but in direct comparisons it has not demonstrated a clear difference in outcomes (33). Additionally, fiber-optics may have advantages over digital ureteroscopy in specific scenarios, such as accessing difficult lower pole calices (42). Despite the technical improvements seen in digital ureteroscopy, more research will be needed to evaluate its effectiveness in UTUC management.

## Percutaneous approaches

Management of UTUC can be done *via* an antegrade percutaneous endoscopic method or a retrograde ureteroscopic method. Percutaneous approaches are primarily performed in patients with large, low-grade disease whose tumors are not amenable to retrograde ureteroscopic treatment. This may be due to the anatomic location of the disease, such as those in the lower pole. Antegrade treatment is also the initial method for patients with existing percutaneous tracts, such as those with urinary diversions. Percutaneous electroresection has been a longstanding method of treatment for UTUC, with a key benefit being its ability to spare the kidney from radical resection in a majority of cases (43–45). An advantage to antegrade access is that large caliber tools such as flexible cystoscopes or rigid nephroscopes can be used following obtainment of access, and biopsy or resection can be completed with cup biopsy forceps, bipolar loop resectoscopes with normal saline irrigation (which obviates risk of electrolyte imbalances associated with hypotonic irrigation), or laser probes (18), with good safety outcomes along with high histologic yield and grade concordance (46). Percutaneous resection or ablation procedures are frequently augmented with adjuvant topical mitomycin chemotherapy or bacillus Calmette-Guerin (BCG) immunotherapy (44, 45, 47).

Though percutaneous procedures are inherently more invasive than retrograde endoscopic counterparts, percutaneous biopsies of UTUC have shown minimal risk of tract seeding and intravesical recurrence when compared against retrograde procedures (46, 48, 49). The process of percutaneous access can provide the additional benefit of facilitating nephrostomy tube placement, which may help treat severe hydronephrosis or impending renal failure for UTUC patients with little seeding risk (49). Conversely, studies of percutaneous resection of UTUC have shown an association of percutaneous resection with recurrence. Across a population of 141 patients accrued over 30 years, Motamedinia et al. in 2016 found a recurrence rate of 37% in low-grade UTUC patients and 63% in high-grade patients who underwent percutaneous resection (44). Similarly, Strijbos and van der Heij in 2016 reported an upper tract recurrence rate of 50% in a sample of 40 UTUC patients who underwent percutaneous tumor resection (45).

Numerous case reports have been written detailing individual patients who experienced seeding of upper tract (50, 51), bladder (52–54), or renal cancers (55) *via* percutaneous nephrostomy tubes. A 2021 systematic review and meta-analysis by Sountoulides et al. found that stenting after resection of bladder tumors was associated with increased risk of metachronous UTUC at a rate of 7.2%. The authors also found no statistical difference between stent and nephrostomy placement for metachronous UTUC development, suggestive of an equivalent risk for both placements (56). However, data from larger studies that are UTUC-specific is limited. In a 2019 comparison of 25 patients with UTUC and percutaneous nephrostomy placement compared to 639 UTUC patients without nephrostomy, Huang et al. found that 20% and 30.8% of patients, respectively, had either local recurrence or distant metastasis, suggesting that percutaneous nephrostomies had little effect on risk of tumor seeding (49). At this time, there remains a scarcity of data with larger populations of UTUC patients who have undergone percutaneous nephrostomy tube placement, and the true effect of percutaneous nephrostomy on seeding risk has yet to be fully characterized.Retrograde endoscopic resection/ablation

Endoscopic resection of UTUC may be performed as part of the initial ureteroscopic tumor visualization and biopsy, while both endoscopic resection and ablation can be curative treatments for low-grade disease. Electrocautery is a longstanding method of endoscopic resection, typically done *via* bugbee fulguration. This method has particular utility for resection at the lower pole calices where laser fibers may not have sufficient down-deflection for complete ablation. Flexible ureteroscopes may also be used for loop electroresection (18, 57). These devices are commonly used for resection of bladder tumors in conjunction with standard tools such as cup biopsy forceps, baskets, and graspers. However, energy settings must be reduced due to increased risk of perforation in the ureter compared to the bladder as well as risk of ureteral stricture formation after fulguration. Electroresection techniques and devices have seen decreasing use in the management of UTUC. This is partly attributable to the previously discussed risks for transmural urothelial injury, as well as the need for hypotonic irrigation with any monopolar resection leading to risk of electrolyte disturbances. Due to their use of normal saline for irrigation, endoscopic laser ablation techniques have largely supplanted retrograde electroresection (57, 58).

Laser probes may be used as part of the hemostasis process following standard resection, or may be the primary tool for resection and fulguration of deeper tumor tissue. Neodymium: yttrium-aluminum-garnet (YAG) and holmium:YAG are the most commonly used laser types in current practice, while thulium:YAG is a newer modality that has shown positive results in early studies. Neodymium:YAG is the oldest laser type still in common use, with initial studies dating to the mid-1980s (59) and early 1990s (60–62). Neodymium:YAG lasers have a wavelength of 1.064 μm and penetration depth of 5 to 6 mm (18). Neodymium:YAG has utility for large (≥ 2 cm) lesions (63), and has superior tissue coagulation effect while destroying target lesions (64). Holmium:YAG has a longer wavelength of 2.1 μm, but less depth of penetration at under 0.4 mm (48). Like neodymium:YAG, it is most effective for large lesions. Studies of laser ablation efficacy have shown increased rates of recurrence compared to radical nephroureterectomy, but with the benefit of renal unit preservation (63). A potential benefit of holmium:YAG is a decreased rate of recurrence in tumors <1 cm versus >1 cm, but radical nephroureterectomy remains the standard method of treatment for high grade disease (65). Neodymium:YAG and holmium:YAG may be applied as a combined modality with individual switch settings (58). This combination has shown good outcomes in treating large, multifocal, low-grade UTUC, with a 93.2% progression-free survival rate (63, 66). For either laser type, surgeons can use 200 to 365 μm fibers to achieve either coagulation or ablation/vaporization of target tissue. Coagulation requires a longer pulse with low energy at 0.5–0.6 J, reduced frequency at 5 Hz, or increased distance from the laser to the target to defocus the beam. Conversely, ablation or vaporization needs a shorter pulse with higher energy and frequency (0.6–1 J at 10 Hz) and a closer distance to the target. When operating in the ureter, ablation is the preferred technique due to decreased length of subsequent strictures (58). Recently, Zou et al. conducted a pilot clinical experience to perform ureteroscopic cryoablation following holmium:YAG ablation. This process involves application of a liquid nitrogen probe on the residual tumor site, leading to formation of an ice ball and induction of necrosis/apoptosis from mucosa to lamina muscularis. This procedure shows early promise as an additional management technique to reduce recurrence following primary laser-based tumor destruction (67).

Thulium:YAG lasers have a wavelength of 2.0 μm and 0.25 mm depth of penetration. In *ex vivo* porcine models, thulium:YAG has shown a lower risk profile compared to holmium:YAG due to decreased depth of incision, larger coagulation area, and larger total laser area (68). In the clinical setting, thulium:YAG has shown good safety and efficacy in recent trials, with recurrence rates of approximately 19% and predominantly minor complications (69, 70). Combination treatment with holmium:YAG and thulium:YAG has also been employed with good efficacy and rates of renal preservation (with PFS exceeding that of holmium:YAG laser treatment alone) (71), as well as in conjunction with photodynamic diagnosis guidance (72). As with percutaneous treatments, endoscopic laser procedures are often augmented with chemotherapy and immunotherapy instillations (13, 73, 74). Recently, thulium has also been deployed in the form of thulium fiber laser (TFL), a super-pulsed laser with a wavelength 1.94 μm and a penetration depth of 0.077 mm, under a quarter of holmium:YAG penetration and with decreased tissue ablation thresholds. TFL requires additional validation, however promising results indicate better energy absorption, limited penetration and limited carbonization, thereby producing a limited ablation zone (75). Laser devices are a continuing area of development, with newer devices such as 1470 nm laser diode (76) and 532 nm green-light laser (77) undergoing preliminary use in clinical settings (76).

## Instillations

Topical instillations of chemotherapy or immunotherapy are frequently used as adjuvant treatment post-resection/ablation, and more recent work has seen the development of new applications in the form of primary therapy. Instillations may be delivered in an antegrade or retrograde direction. Antegrade delivery is typically administered *via* a 10 Fr nephrostomy tube placed percutaneously, and is therefore performed in cases where resection/ablation was achieved *via* percutaneous access. The antegrade approach has several risks associated with percutaneous access, including risk of bacterial colonization due to the exposed system with potential development of sepsis and leakage of the administered agent alongside the nephrostomy tube (73). Retrograde delivery can be attained with placement of a 5 Fr cystoscopic ureteral catheter or single-J stent, however this method requires cystoscopy and catheter placement at each visit for subsequent instillations. A double-J stent can be placed once and accessed *via* ureteral catheter on subsequent visits without necessitating reinsertion of the ureteral stent. Though this eliminates the need for catheter placement on subsequent visits, there is a risk of reduced reflux leading to insufficient agent delivery and dwell time (78). Pollard et al. compared the efficacy of delivery *via* antegrade nephrostomy, indwelling double-pigtail stent with reflux, and 5F open-ended ureteral catheter with retrograde infusion in an *ex vivo* porcine model with nine renal units (3 per approach). The authors compared percent area of urothelial surface stained by indigo carmine instillation in each method, and found that retrograde instillation with open-ended catheter produced the greatest staining with 83.5% of total area stained (compared to 65.2% for antegrade nephrostomy and 66.2% for indwelling stent; p=0.002). These results suggest retrograde infusion is the most effective instillation approach, but larger studies with *in vivo* models are needed for further validation (79).

The most common use of instillations is subsequent to resection/ablation procedures, with the goal of reducing intracavitary recurrence of disease. Adjuvant chemotherapy is given in patients with low- to intermediate-risk disease, though immunotherapy with BCG may also be given instead of or in addition to chemotherapy (13, 18). The most frequently used chemotherapy agent is mitomycin C. Oher chemotherapies that have seen use in clinical settings include pirarubicin (80, 81), epirubicin, gemcitabine (82), and adriamycin (83). Adjuvant immunotherapy is most commonly *via* BCG, though it has also been demonstrated with combined BCG and interferon-α2B (84). Adjuvant BCG may be administered to patients with low/intermediate-risk disease. High-risk disease, including UTUC *in situ*, may also be managed with BCG instillation as a primary treatment, but not as adjuvant therapy (18). A 2019 meta-analysis by Foerster et al. found that the choice of drug or delivery approach has little effect on rate of recurrence, which is approximately 40% for stage Ta/T1 UTUC treated with either BCG, mitomycin, a combination of the two, and 32% for *in situ* disease following BCG treatment only (13). Physicians might best determine treatment decisions, then, according to individual patient characteristics and comorbidities in conjunction with choice of drug or delivery approach. Regardless of treatment choice, adjuvant instillations have demonstrated strong positive benefits for UTUC patients, leading to their inclusion in European Associations of Urology guidelines as recommended steps in perioperative management of UTUC. However, instillations have yet to see broad adoption by many clinical practices (85). The exact causes of this discrepancy remain an ongoing area of investigation.

Intravesical instillations as primary treatment for UTUC is an increasing area of investigation. Instillation of BCG using reflux *via* double-pigtail catheter was established as a primary treatment for upper tract carcinoma *in situ* in the early 2000’s, particularly in patients ineligible for surgery (86, 87). Progression rates for *in situ* disease primarily treated with BCG may be as low as 5% (88). In 2017, Metcalfe et al. reported on the use of mitomycin C as both induction and adjuvant therapy to either percutaneous nephrostomy or cystoscopic ureteral catheter treatment of Ta/T1 UTUC, with three-year recurrence-free, progression-free, and nephroureterectomy-free survival rates of 60%, 80%, and 76% (73). Mitomycin C has also seen increased utilization in the form of Jelmyto, a reverse thermal gel formulation approved by the U.S. Food and Drug Administration in April 2020 (89). As established in the OLYMPUS trial, Jelmyto can be administered for treatment of low-grade UTUC *via* retrograde 5–7 Fr catheters. The initial administration is in liquid form, which converts to semi-solid gel after instillation at the target. This gel is then dissolved by urine flow over 4–6 hours (90). Final trial results in 2022 showed a 58% complete response to induction, with 59% complete response after maintenance induction and 50% complete response without maintenance induction. Ureteric stenosis was the most common treatment-emergent adverse event (91). Building on this work, Rosen et al. described a technique for antegrade Jelmyto instillation *via* percutaneous nephrostomy tube with comparable response rate to the originally established retrograde technique. This approach obviates need for repeated ureteroscopy and fluoroscopy, and may reduce the risk of instrumentation-induced ureteral stricture. Small samples, however, prevent any conclusions regarding superiority of antegrade or retrograde Jelmyto delivery at this time (89). Gel formulations of other therapy agents are an area of active investigation, with Kesch et al. evaluating instillation of a mucoadhesive paste formulation of gemcitabine in a preclinical *in vivo* porcine trial (92).

There is limited data on the efficacy of intracavitary instillations as second-line or salvage therapy for UTUC. Balasubarmain et al. investigated response to second-line and salvage therapy with topical instillations following recurrence after primary endoscopic treatment. Across 18 renal units receiving second-line treatment (10 receiving treatment as reinduction, 8 receiving treatment as salvage therapy), 5 of the 18 renal units had carcinoma *in situ* that was unresponsive to initial topical therapy and received salvage topical therapy with either mitomycin C or BCG, while the remaining 13 renal units had recurrent or relapsing papillary tumors and received salvage therapy with mitomycin C, BCG, gemcitabine, or mitogel. Carcinoma *in situ* salvage response rates were low at 1 of 5 patients (20%), while response rates for salvage therapy for papillary tumors was 8 out of 13 patients (61.5%). Their results suggest a potential role for salvage therapy in low-risk disease, but given the small study size, any change in guidelines will be secondary to validation with larger groups and standardized treatments (93).

## Conclusion

The role of endoscopy in management of UTUC has greatly expanded over the 21^st^ century. Endoscopic management can encompass both diagnostic and therapeutic steps in management, spanning visualization, resection, and instillation techniques. Endoscopic management has strong outcomes as treatment for low-risk disease. As devices, techniques, and indications continue to expand, continuing studies will be needed to establish the continually evolving best clinical practices. Furthermore, given the potential long-term benefits of preserved renal function, nephron-sparing procedures should be considered for all patients with non-invasive tumors. The wide variety of approaches to endoscopic management enables providers to select methods as best fits individual patient profiles and provider skillsets. In light of this customizability and strong outcomes in curative management across several recent studies, endoscopic management should be considered a viable option for all patients with non-invasive tumors regardless of size or macroscopic architecture.

## Figures and Tables

**TABLE 1:** Summary of Imaging and Biopsy Methods.

Diagnostic Method	Study	Mechanism
Endoluminal Ultrasonography (ELUS)	Farnum et al (2018) (26)	A radial scanning ultrasound at 20 MHz in B-mode with a 5F probe is used to visualize the upper tract.
Narrow-Band Imaging (NBI)	Traxer et al (2011) (27)	White light is filtered into blue and green wavelengths that better penetrate mucosa and are absorbed by hemoglobin, thereby forming a more detailed view of mucosal tissue and blood vessels.
Photodynamic Diagnosis (POD)	Qiangzhao et al (2020) (28)	A photosensitizing agent is injected into the intravesical cavity or administered orally. During endoscopy, blue light (375–445 nm) is shone causing the agent to fluoresce.
Optical Coherence Tomography (OCT)	Ikeda et al (2013) (29); Bus et al (2014) (29)	Back-scattered light produces micrometer-scale resolution cross-sectional images.
Confocal Laser Endomicroscopy (CLE)	Chen et al (2014) (30); Breda et al (2018) (31)	A 488nm low-energy laser scans tissue stained with fluorescein in real-time. An intraoperative photosensitizer excites the stained tissue, which emits light that is filtered so only in-focus light is recorded, leading to high resolution intraoperative imaging and potential tumor grading.
Digital Ureteroscopy	Gridley and Knudsen (2017) (32); Soria et al (2021) (33)	Photons are converted to electrons that are carried *via* digital signals to an image processor which produces a real time view.
